# Dr. Abercrombie on a Peculiar Affection of the Pericranium, Relieved by Division of the Membrane

**Published:** 1830-01-01

**Authors:** 


### XLVI.

### PECULIAR AFFECTION OF THE PERICRANIUM, ACCOMPANIED WITH REMARKABLE SYMPTOMS, AND GENERALLY RELIEVED BY DIVISION OF THE MEMBRANE.

Dr. Abercrombie, in the second edition of his valuable work on the Diseases of the Brain and Spinal Cord, has an interesting chapter on a peculiar affection of the pericranium, occasionally met with in practice. Sir Everard Home was, we believe, the first who gave an accurate description of the disease, and Dr. Abercrombie has furnished such a neat abrégé of his opinions, that we cannot do better than copy the latter.

"In the cases related by Sir Everard Home,* the symptoms in general were headach, with various uneasy feelings in the head, and a painful tenderness of the scalp at a particular spot, with some degree of swelling or thickening of the integuments at the place. In one, the sight and hearing were considerably impaired; and in several of the cases there were fits resembling epilepsy. They were treated by dividing the integuments and pericranium freely down to the bone, and then dressing the wounds with lint, so as to allow them to heal slowly with suppuration. In making the incision, the pericranium was found morbidly sensible, and considerably thickened; and in some of the cases indurated, approaching to the structure of cartilage. This treatment was in some of them followed by immediate and permanent relief; in others, the patient continued liable to fits or head symptoms upon any excess. In some of them, the incisions healed without any affection of the bone being discovered; in others, a portion of the bone appeared white and porous, or honey-combed, and a limpid fluid appeared to percolate through it, which returned immediately as often as it was wiped off. In one of these cases, the porous piece of bone exfoliated after the wound had been dressed with dry lint for six weeks; the wound then healed, and the cure was permanent. In another, after waiting eight weeks for the exfoliation, he touched it repeatedly with diluted nitric acid, after which it exfoliated, and the cure was permanent. In one fatal case, he found the pericranium thickened into a mass of a fibrous bony texture, and, corresponding to this part internally, there was a similar thickening and induration of the dura mater. Most of these cases had been treated by long courses of mercury without benefit, in some of them with aggravation of the symptoms."

Mr. Crampton has described a disease somewhat similar in many respects, under the head of periostitis. It may be remarked, however, that Mr. C.'s cases do really resemble common inflammation of the periosteal tissue more than Sir Everard Home's, although there is a family resemblance between them.

"Among Mr. Crampton's cases, affecting various parts of the body, there

---

* "Transactions of a Society for the Improvement of Medical and Surgical Knowledge, vol. iii."

are two remarkable examples of it in the head; the one acute, the other chronic. In the former, a boy of 14, the complaint began with a small angry tumour on the right side of the nose, from which, after some days, a swelling extended along the right eyelids and forehead, with considerable erysipelatous inflammation and fever. On the ninth day, he became suddenly comatose, then convulsed, and died on the 12th. On dissection, the pericranium covering the frontal bone was found red, thickened and detached from the bone, much purulent matter lying between them. Internally the dura mater was detached to an extent corresponding to the external disease, and a greenish puriform fluid was effused between it and the bone. The inner surface of the dura mater was also covered with pus; the pia mater was red, very vascular, and covered with pus to the extent of two inches on the part corresponding to the principal disease of the pericranium. The other case is that of a woman, aged 32, who was affected with a tumor the size of a walnut over the left parietal bone. It was soft and elastic, and its origin was ascribed to a blow six months before; there was an opening in the tumour, by which a probe could be passed down to the bone. She had intense pain in the left side of the head; the right arm was wasted and paralytic, and the fingers were contracted; both lower extremities were feeble; her speech was indistinct; she had vomiting, and frequent epileptic fits. The tumour was divided freely down to the bone, and in doing so the pericranium was found thickened, firm, fibrous, and morbidly sensible. It formed the principal part of the tumour. The bone under the tumour was found rough and superficially carious. A portion of it was removed by the trephine, and the dura mater under it appeared very vascular, and rather thickened. For six days after the operation she had fever, extensive erysipelas of the head, delirium, and convulsions. Suppuration was then established, and all these symptoms were relieved. In the course of the cure a slough was detached from the dura mater. A fortnight after the operation she recovered the use of her arm, and was free from complaint."

Tissot, Ponteau, and others describe cases which bear some resemblance to those of Sir E. Home and Mr. Crampton, but the likeness may be fanciful, the comparison unsafe, and we therefore pass on to an instance related by Dr. Abercrombie himself.

*Case.* "A servant girl, aged about 20, fell backwards with a child in her arms, and received the full force of the fall upon the most prominent part of the occipital bone. She soon recovered from the immediate effects of the injury, but continued to have pain in the part; and after several months, was seized with paraplegia and retention of urine. She was now confined to bed for three or four months, after which she recovered the use of her limbs in a tolerable degree, but the retention of urine continued, and she came to Edinburgh in the beginning of 1828, which was more than a year after the accident. The paraplegia was now nearly removed, but she had still retention of urine, requiring the constant use of the catheter. On the seat of the injury on the occipital bone, a round portion, the size of a crown piece was acutely tender, and very moderate pressure upon it produced complete insensibility, which continued a minute or two, and returned as often as the pressure was repeated. It had the appearance of syncope, but the pulse was not affected. In this state I saw her along with Mr. Lizars, and it was agreed to make a free crucial incision through the part, and to keep the wound open by dressings so as to promote suppuration. In doing so, the pericranium was found tender and somewhat thickened, but the bone was sound. On the following day she passed her urine freely, and she continued free from complaint as long as the wound continued to discharge. It healed at the end of a fortnight, and the retention of urine returned immediately. The incision was now repeated with the same result as before, her urine being freely passed almost immediately. Various means were then employed to promote a more complete suppuration from the wound, but it healed after two or three weeks, and the retention of urine returned as before, with considerable tenderness in the affected spot. A third incision was then made with the same effect as before, and various applications were made with the view of promoting exfoliation of bone, as in Sir Edward Home's cases, but without success, and the wound again healed after three or four weeks. The fits of insensibility on pressure now returned, which had not returned after the former incisions, and along with them the retention of urine.

"Since that time repeated incisions have been made with similar results. The principal change in her situation now is, that she has got free of the fits of insensibility upon the spot being pressed; and the effect of the incisions has continued longer, as on several occasions she has remained free from the retention of urine for several weeks after the incisions were healed, and at one time enjoyed perfect health for three months."

We must confess that we are not without our misgivings on the real nature of the foregoing case. The age of the patient, the progress of the symptoms, the retention of urine, and the syncope without affection of the pulse, are features that look but too like those of hysteria, not to stagger our belief in the existence of organic disease. We have seen, and every practical person has seen also, worse symptoms than these dependent on the Proteian disease in question, and we verily believe that retention of urine and pain in the head aggravated by the slightest touch are amongst the more common of its forms. Of course we do not venture to pronounce that Dr. Abercrombie's case *was* one of hysteria, but we think that reasoning on the published data, always inferior to personal examination, its aspect is suspicious.

We remember having witnessed a case of the kind described by Sir Everard Home, some years ago at St. George's Hospital. It was that of a widow, about 30 years of age, who presented some tumefaction over the left temporal ridge of the parietal bone, very tender upon pressure, accompanied with dimness of vision, much pain shooting to the opposite side of the head, disposition to slight vertigo, and numbness of the hands. There was an odd expression about the eyes and aspect which had rather a maniacal cast; her general health was pretty good. She dated her complaints to a severe blow upon the part received seven years previously, which stunned her at the time, and since the infliction of which she had suffered more or less from the symptoms we have enumerated. Mr. Brodie, under whose care the patient was, cut down upon the tumefaction and divided the pericranium. There was no perceptible thickening of the latter, but the bone appeared to Mr. B. to be somewhat enlarged. Several attacks of erysipelas of the face and head succeeded the operation, and it was long before she was able to quit the house. The pain in the head was certainly relieved, but we did not perceive much difference in other respects, and a few days ago, when we casually saw the individual, she was still suffering from swimming in the head, disposition to irregular flushings of the face, and other symptoms of an unpleasant character.

In a clinical lecture delivered on the occasion, Mr. Brodie mentioned one or two interesting cases which he had treated with success. A man became affected with pain in the forehead, and a tumour appeared. A fit of epilepsy succeeded, other epileptic attacks occurred at intervals, and three months after the commencement of the disease he entered St. George's Hospital. There was now a considerable tumour in the forehead; Mr. Brodie cut down upon it; and, finding that it looked like the affection of the pericranium resulting from scrofulous inflammation, he removed it entirely from the bone, which was rough. The pain in the head was relieved, a layer of bone exfoliated, and the patient got quite well. In another instance a woman received a blow upon the head, and ever afterwards suffered from pain in the part which was slightly tumefied. She entered St. George's Hospital complaining of pain in the head, dimness of vision, numbness of one hand, &c.; Mr. Brodie divided the pericranium, which was a little thickened, down to the bone; and this patient also recovered perfectly.

---

